# Natural product based approaches to overcome *Candida glabrata* and emerging AMR threats

**DOI:** 10.3389/frabi.2026.1767032

**Published:** 2026-02-10

**Authors:** Binaya Krushna Sahu, Sudipta Kumar Patra, Mahesh Chandra Sahu, Sujogya Kumar Panda

**Affiliations:** 1 Centre for Biotechnology, Siksha ‘O’ Anusandhan (Deemed to be University), Kalinganagar, Bhubaneswar, Odisha, India; 2 Department of Orthopedics, Kalinga Institute of Medical Sciences, Bhubaneswar, Odisha, India; 3 Division of Microbiology, Indian Council of Medical Research (ICMR)-Regional Medical Research Centre, Chandrasekharpur, Bhubaneswar, Odisha, India

**Keywords:** antifungal resistance, biofilm inhibition, *Candida glabrata*, natural products, phytochemicals

## Abstract

The rise of *C. glabrata* as a serious, multidrug-resistant organism poses a significant and global challenge to the human health. The reasons *C. glabrata* has developed resistance to standard antifungal drugs, include the activation of efflux pumps, the production of biofilms, and changes in ergosterol biosynthesis. In light of the threat posed by *C. glabrata*, the potential of phytochemicals as therapeutic alternatives should be considered due to their diverse structures and ability to exhibit more than one type of antifungal activity. This review summarizes advances in the use of plant-based natural products displaying antifungal activity against *C. glabrata*, with an emphasis on key classes of phytochemicals, including flavonoids, terpenoids, phenolic compounds, alkaloids, and essential oils. While the proposed mechanisms include disruption of cell membranes, inhibition of ergosterol synthesis, attenuation of oxidative stress, and suppression of virulence and biofilm formation, it is important to note that most evidence arises from *in vitro* studies, with only limited mechanistic investigations on individual compounds. Although *in vitro* studies indicate promising antifungal and adjunctive effects, the available evidence remains largely preclinical, with variable synergistic outcomes. Such synergy not only enhances therapeutic efficacy but also reduces required drug dosages, thereby minimizing toxicity and delaying the emergence of resistance. Major limitations include inconsistency in phytochemical composition, insufficient pharmacokinetic data, and a lack of robust *in vivo* and clinical studies. This review critically integrates current knowledge, highlighting both the multi-target potential of phytochemicals against *C. glabrata* and the key challenges that must be addressed to enable realistic clinical translation. By prioritizing synergy-focused research, and methodological standardization, phytocompounds can be positioned not merely as standalone agents but as adjunctive modulators of antifungal resistance, paving the way for novel, effective, and sustainable therapeutic options against MDR *C. glabrata*.

## Introduction

Globally, invasive fungal infections pose a serious health issue, particularly for individuals who are hospitalized or immunocompromised. *Candida* species remain the most common cause of both superficial and systemic infections among fungal pathogens (Das et al., 2026). The epidemiology of candidemia has undergone significant changes over the past two decades. Previously, most cases were caused by *Candida albicans*, but non-albicans species, particularly *C. glabrata* (recently also known as *Nakaseomyces glabratus*), are now more frequently implicated (Katsipoulaki et al., 2024; Mallick et al., 2025; Sahu et al., 2025). Recent surveillance studies consistently report that *C. glabrata* accounts for approximately 13-30% of candidemia cases globally, with higher prevalence in hospital settings and high-risk patient populations, underscoring its growing clinical relevance (Jenkins, 2025; Dai et al., 2025; Yamin et al., 2025).

*C. glabrata* propensity for decreased sensitivity or complete resistance to traditional antifungal treatments increases its therapeutic importance. Fluconazole resistance rates commonly range from 2.6% to 10.6%, but can exceed 17% in certain regions, while echinocandin resistance, though generally low that has been increasingly reported in specific hospital settings (Won et al., 2021; https://www.cdc.gov/candidiasis/data-research/facts-stats/, https://www.cdc.gov/candidiasis/antimicrobial-resistance/index.html).

The prevalence of an increasing proportion of multidrug-resistant (MDR) *C. glabrata* is a serious public health issue due to the limited number of antifungal drug types and the high costs associated with treatment (Hassan et al., 2021). In fact, there is a significant global burden of invasive candidiasis: hundreds of thousands of cases of *Candida* bloodstream infection (BSI) are thought to occur each year, and these cases are frequently linked to high rates of morbidity and mortality (Rabault et al., 2025; Kotey et al., 2021; https://www.cdc.gov/candidiasis/antimicrobial-resistance/index.html).

These trends underscore the urgent need for innovative antifungal drugs with novel mechanisms of action, enhanced safety profiles, and a reduced propensity to induce resistance. In this context, plant-derived natural products, collectively referred to as phytochemicals, offer an attractive and underexplored reservoir of antimicrobial compounds. Many modern pharmaceuticals originate from natural products, and phytochemicals are characterized by structural diversity and the ability to target multiple fungal pathways simultaneously (Soliman et al., 2017; Dong et al., 2023).

Many classes of phytochemicals, such as flavonoids, terpenoids, alkaloids, phenolic acids, and components of essential oils, have been shown in recent reviews to exhibit potent antifungal activity against a variety of *Candida* species both *in vitro* and, in certain situations, *in vivo* (Esmaeili et al., 2025; Lu et al., 2017; Kerkoub et al., 2018; Kipanga et al., 2020). These natural substances have antifungal effects by interfering with virulence traits like biofilm formation and adhesion, disrupting fungal cell membranes, inhibiting ergosterol or other cell-wall or cell membrane biosynthesis pathways, inducing oxidative stress, and impairing efflux pump activity (Dantas et al., 2025; Khwaza and Aderibigbe, 2023; Esmaeili et al., 2025; El-Saadony et al., 2025; Panda et al., 2023; Soliman et al., 2017) (**Table 1**).

**Table 1:** Major classes of phytochemicals with demonstrated activity against *Candida glabrata*.

Phytochemical class	Representative compounds/Sources	Primary antifungal mechanisms	Evidence against *C. glabrata*	References
Flavonoids	Quercetin, EGCG, Catechin, Apigenin	Efflux pump inhibition; disruption of ergosterol biosynthesis; ROS induction; antibiofilm and anti-adhesion activity	Inhibits azole-resistant isolates; reduces biofilm biomass; restores fluconazole susceptibility	Al Aboody and Mickymaray, 2020; Chen et al., 2015; Hervay et al., 2023; Schwarz et al., 2022
Phenolic Compounds	Gallic acid, Caffeic acid, Ellagic acid, Propolis extracts	Membrane destabilization; ROS-mediated oxidative stress; ECM disruption; metal ion chelation affecting biofilm stability	Strong antibiofilm activity; synergistic with azoles; effective against planktonic and biofilm cells	Teodoro et al., 2015; Alves et al., 2014; Li et al., 2015; Sampaio et al., 2021; Kerkoub et al., 2018; Szweda et al., 2015; Fernandez-Calderon et al., 2021; Sampaio et al., 2021; Wicaksono et al., 2020
Terpenoids	Thymol, Carvacrol, Geraniol, Ursolic acid, Betulinic acid	Membrane permeabilization; mitochondrial dysfunction; inhibition of cell wall synthesis; biofilm disruption	Fungicidal activity against resistant strains; effective against persister cells; weakens echinocandin tolerance	Ahmad et al., 2011; Haque et al., 2016; Gupta and Poluri, 2021; Marena et al., 2021; Tan et al., 2024
Alkaloids	Berberine, Piperine, Tetrandrine	Efflux pump inhibition; mitochondrial dysfunction; interference with nucleic acid synthesis	Overcomes PDR1-associated resistance; effective alone and in combination therapies; antibiofilm effects	Dhamgaye et al., 2014; Gupta et al., 2023; Ding et al., 2025
Essential Oils	Oregano oil, Thyme oil, Cinnamon oil	Cell membrane disruption; ergosterol depletion; quorum-sensing interference; antibiofilm activity	Potent activity against azole-resistant strains; reduces biofilm biomass; synergistic with azoles and echinocandins	Bakkali et al., 2008; Fernandes et al., 2023; Shreaz et al., 2011

The increasing incidence of infections caused by non-*albicans Candida* species, including *C. glabrata*, particularly in light of their growing clinical importance and drug resistance patterns, highlights an urgent need to explore natural products for antifungal activity against these organisms. Much of the current research on antifungal natural products has thus far focused primarily on *C. albicans* (Dong et al., 2023; Calegari-Alves et al., 2025; Katsipoulaki et al., 2024) (**Table 2**). This review focuses specifically on plant-based compounds that can fight *C. glabrata*, a fungal pathogen that is often resistant to common antifungal drugs and less studied than other *Candida* species. It clearly explains how these natural compounds work, including their ability to reduce biofilms and their potential to enhance the effect of existing antifungal medicines. We aim to catalog key classes of phytochemicals with confirmed antifungal efficacy, critically identifying potential mechanisms through which they exert antifungal activity and evaluating available evidence for adjunctive or synergistic interactions between plant-derived preparations and known antifungal drugs, while also acknowledging current limitations and research gaps.

**Table 2:** Mechanisms of antifungal resistance in *Candida glabrata* and corresponding phytochemical interventions.

Resistance mechanism in *C. glabrata*	Molecular features or clinical consequence	Phytochemical counteraction	How phytochemical acts	Reference
Efflux pump overexpression	Upregulation of ABC (CgCDR1, CgCDR2) and MFS (SNQ2) → reduced intracellular azole levels; often PDR1 GOF driven	Flavonoids/alkaloids (quercetin, berberine, EGCG)	Inhibit efflux activity or downregulate transporter expression; restore intracellular azoles	Dhamgaye et al., 2014; Al Aboody and Mickymaray, 2020; Gupta et al., 2023
PDR1 gain-of-function (GOF) mutations	Constitutive activation of multidrug resistance regulon → high-level azole resistance	Berberine, quercetin (as adjuvants)	Inhibit downstream efflux activity; phenotypic reversal of resistance *in vitro*	Dhamgaye et al., 2014; Gupta et al., 2023
Altered ergosterol biosynthesis	Mutations or regulation in ERG genes (ERG11, ERG3) → reduced azole binding/altered membrane sterols	Phenolics/flavonoids (ellagic acid, apigenin, gallic acid)	Interfere with sterol biosynthesis or deplete ergosterol content	Alves et al., 2014; Li et al., 2015; Sampaio et al., 2021
Echinocandin (FKS) hotspot mutations	Point mutations in FKS1/FKS2 → reduced echinocandin binding	Membrane-active terpenoids (thymol, carvacrol)	Kill via membrane permeabilization independent of β-1,3-glucan synthase	Ahmad et al., 2011; Haque et al., 2016
Biofilm matrix sequestration & diffusion barrier	ECM (β-glucans, eDNA, proteins) reduces drug penetration; presence of persisters	Essential oils & phenolics (cinnamaldehyde, oregano oil, gallic acid, EGCG)	Disrupt matrix, degrade/extract β-glucans, increase drug penetration	Kerkoub et al., 2018; Fernandes et al., 2023; Shreaz et al., 2011
Persister cells and metabolic dormancy in biofilms	Subpopulation tolerant to antifungals despite genotypic susceptibility	Terpenoids & alkaloids (carvacrol, thymol, berberine) in nanoformulations	Penetrate biofilms and deliver sustained exposure that kills persisters	Gupta and Poluri, 2021; Essid et al., 2023
Upregulated stress responses (oxidative, heat shock)	Enhanced ROS detoxification (Yap1, Hsp90 pathways) → survival under antifungal stress	Phenolic acids, flavonoids (caffeic acid, EGCG, quercetin)	Modulate redox balance, induce ROS beyond tolerance → apoptosis-like death	Teodoro et al., 2015; Al Aboody and Mickymaray, 2020
Upregulated adhesion (EPA family) promoting persistence	Increased adhesion fosters biofilm formation and device colonization	Flavonoids & phenolics (naringenin, apigenin, gallic acid)	Downregulate adhesin expression and reduce adhesion/biofilm initiation	Sampaio et al., 2021; Al Aboody and Mickymaray, 2020
Overexpression of drug-modifying enzymes or detoxifiers	Increased metabolism or sequestration of antifungals	Tannins/high-MW phenolics (propolis extracts, ellagitannins)	Bind/inactivate extracellular drug or matrix components, enhance local antifungal concentration	Kerkoub et al., 2018; Fernández-Calderón et al., 2021
Efflux-independent reduced drug uptake	Altered membrane composition decreases passive drug diffusion	Membrane-active terpenoids & essential oils (thymol, carvacrol, eugenol)	Increase membrane fluidity/permeability to facilitate drug influx	Bakkali et al., 2008; Ahmad et al., 2011
Quorum sensing–mediated tolerance	QS molecules regulate biofilm maturation and drug tolerance	Phenolics/EO constituents (cinnamaldehyde, rosemary constituents)	Interfere with QS signaling and block biofilm maturation	Shreaz et al., 2011; Fernández-Calderón et al., 2021

## 2 Biological characteristics of *C. glabrata* relevant to antifungal resistance

### 2.1 Genomic and physiological features

**Figure 1:**
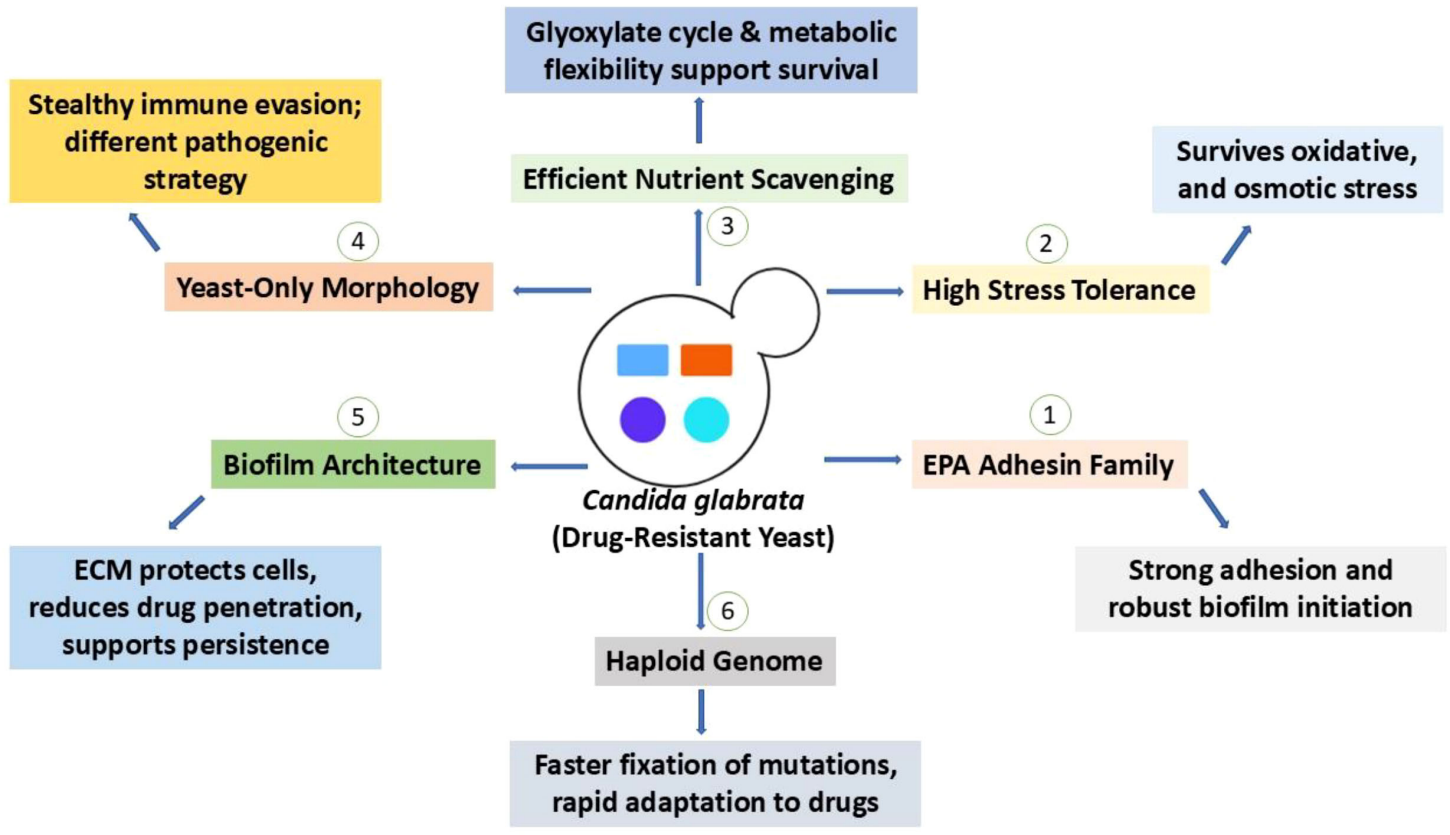
Biological features of *Candida glabrata* contributing to antifungal resistance and persistence. The figure summarizes key genetic, physiological, and virulence-associated traits that collectively enhance survival and multidrug resistance in *C. glabrata*. (1) The EPA (Epithelial Adhesin) family mediates strong adhesion to host tissues and abiotic surfaces, promoting robust biofilm initiation. (2) High stress tolerance enables survival under oxidative, osmotic, and other environmental stresses encountered during host infection and antifungal exposure. (3) Efficient nutrient scavenging and metabolic flexibility, including reliance on alternative carbon utilization pathways, support persistence under nutrient-limited conditions. (4) A yeast-only morphology contributes to stealthy immune evasion and a pathogenic strategy distinct from dimorphic *Candida* species. (5) Complex biofilm architecture, with an ECM, protects embedded cells, limits antifungal penetration, and supports long-term persistence. (6) The haploid genome facilitates rapid fixation of resistance-conferring mutations, accelerating adaptation to antifungal drugs. Together, these features reduce antifungal susceptibility and promote the development and maintenance of multidrug resistance in *C. glabrata*.

Phylogenetically and functionally, *C. glabrata* differs from the polymorphic *C. albicans* (Katsipoulaki et al., 2024). *C. glabrata* is a haploid organism, in contrast to the majority of pathogenic *Candida* species, and genome sequencing and comparative genomics place it significantly closer to the baker’s yeast *Saccharomyces cerevisiae* than to *C. albicans* (Roetzer et al., 2011; Enkler et al., 2016). The haploid genome enables the faster fixation of adaptive mutations under pharmacological selection and provides a genetic background that can more quickly reveal the phenotypic effects of single-nucleotide modifications (Roetzer et al., 2011; Hassan et al., 2021). *C. glabrata* and *C. albicans* differ physiologically in several therapeutically significant aspects that impact antifungal susceptibility, including diminished filamentation capacity. *C. glabrata* often only develops as budding yeast (blastoconidia), without the strong hyphae/pseudohyphae production characteristic of *C. albicans*. This “yeast-only” lifestyle alters host interaction and immune recognition, influencing dissemination strategies (Roetzer et al., 2011). High stress tolerance of *C. glabrata* exhibits pronounced tolerance to oxidative, nitrosative, and osmotic stresses encountered in the host (macrophage oxidative burst), mediated by a compact set of stress-response regulators (Kaloriti et al., 2012; Raj et al., 2024). This ability contributes indirectly to treatment failure and permits survival in challenging microenvironments. Strong adherence to host epithelia and abiotic surfaces is mediated by adhesins of the EPA (epithelial adhesin) family; many EPA genes are subtelomeric and variably expressed, allowing for quick phenotypic switching in adhesiveness (Timmermans et al., 2018; Fernández-Pereira et al., 2021). One of the primary factors influencing tolerance is adhesion, the initial stage of biofilm formation. Effective nutrient scavenging and metabolic adaptability, *C. glabrata* has developed effective absorption and utilization pathways that enable survival in biofilm environments and under nutrient constraint (Raj et al., 2024; Enkler et al., 2016). These physiological and genetic characteristics work together to produce a pathogen that is highly adaptive and stealthy, characteristics that promote persistence throughout antifungal therapy (**Figure 1**).

Importantly, these distinctive genomic and physiological traits also define critical therapeutic vulnerabilities. The reliance of *C. glabrata* on stress-response pathways, membrane plasticity, adhesion-mediated biofilm initiation, and metabolic flexibility provides multiple entry points for phytochemicals, which often exert multitarget effects rather than acting on a single molecular site. As discussed in later sections, several plant-derived compounds exploit these vulnerabilities by disrupting membrane integrity, impairing stress tolerance, and inhibiting adhesion and biofilm establishment, thereby counteracting the adaptive advantages conferred by these biological features.

### 2.2 Mechanisms of antifungal resistance

Resistance in *C. glabrata* is multifactorial and often arises during therapy. The main mechanisms implicated in clinical isolates are:

#### 2.2.1 Efflux pump overexpression

**Figure 2:**
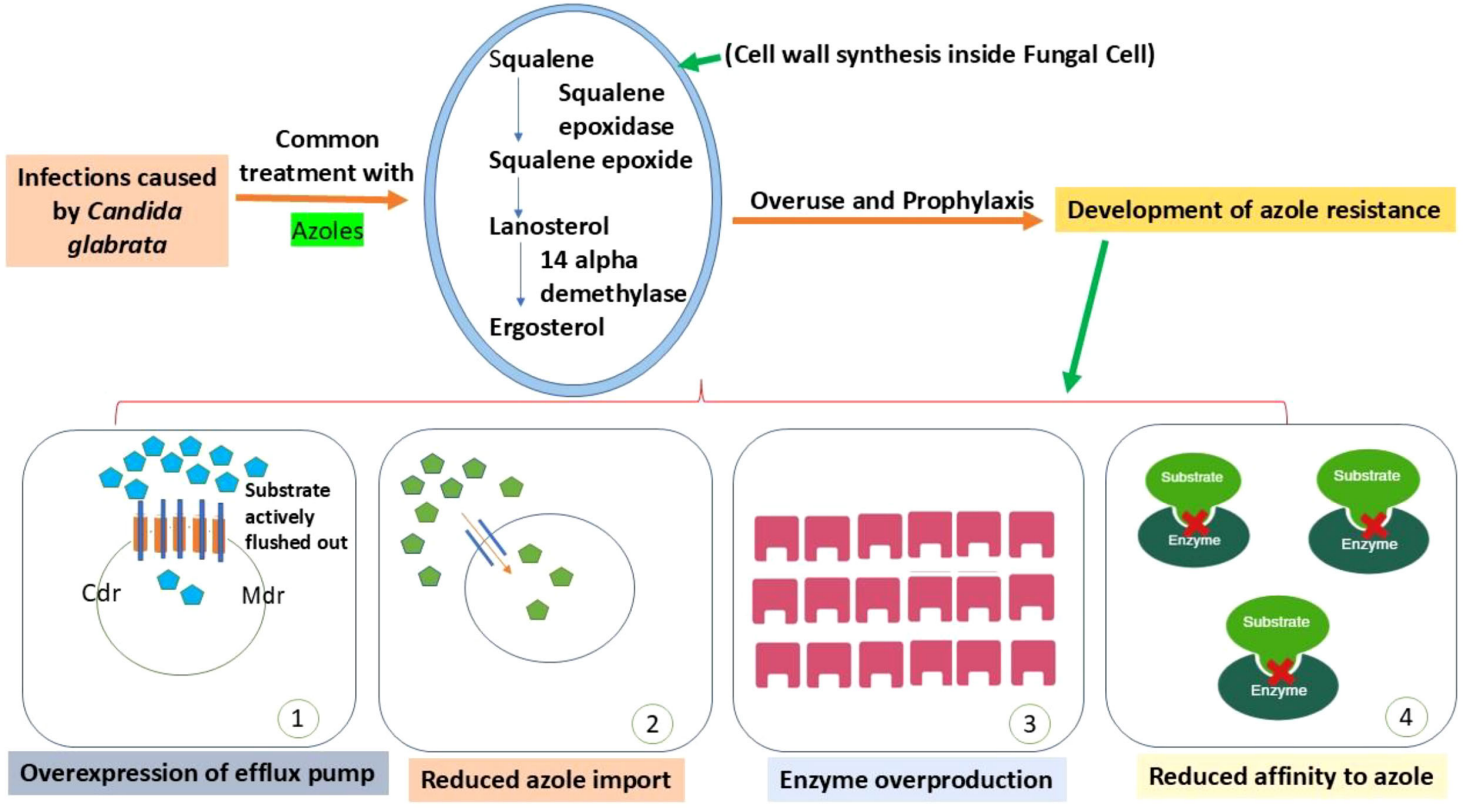
The primary mechanisms that contribute to azole resistance in *C. glabrata*. Azole drugs target the ergosterol biosynthesis pathway by inhibiting 14-α-demethylase, disrupting fungal cell membrane formation. However, repeated exposure, overuse, and prophylactic treatment drive the development of azole resistance. *C. glabrata* employs several adaptive mechanisms to evade azole activity: (1) Overexpression of efflux pumps such as Cdr (ABC transporters) and Mdr (MFS transporters), which actively expel azole molecules from the cell; (2) Reduced azole import, limiting intracellular drug accumulation; (3) overproduction or functional alteration of sterol biosynthesis enzymes, including Erg3, Erg5, and Erg6, in addition to 14-α-demethylase (Erg11), which counteracts the inhibitory effect of azoles; and (4) Reduced azole affinity, caused by alterations in the drug target enzyme, preventing effective binding. Together, these mechanisms significantly reduce azole susceptibility and promote multidrug resistance in *C. glabrata*.

Major facilitator superfamily (MFS) and ATP-binding cassette (ABC) transporters, particularly CgCDR1, CgCDR2, and CgSNQ2, are often overexpressed in clinical and experimental isolates. These transporters actively pump azoles and other compounds out of the cell, reducing intracellular drug concentrations (Sanguinetti et al., 2005; Torelli et al., 2008). Gain-of-function (GOF) mutations in the transcription factor PDR1, which up regulate several drug-resistance genes and have been frequently connected to the failure of fluconazole therapy, are frequently the cause of overexpression (Castanheira et al., 2022; Sanguinetti et al., 2005) (**Figure 2**).

#### 2.2.2 Alterations in ergosterol biosynthesis and membrane composition

Even though ERG11 mutations are less frequently the leading cause in *C. glabrata* than in *C. albicans*, changes in the genes involved in the ergosterol pathway and regulatory modifications that change the amount of sterol in the membrane have been noted and can either mediate cross-resistance to polyenes or decrease azole susceptibility (Vandeputte et al., 2012; Vu and Moye-Rowley, 2022; Memon et al., 2025). Crucially, sterol homeostasis and membrane composition may be indirectly impacted by PDR1-mediated alterations, exacerbating resistance mechanisms.

#### 2.2.3 Echinocandin resistance via FKS mutations

β-1,3-glucan synthase is the target of echinocandins. The leading molecular cause of acquired echinocandin resistance in this species is point mutations in conserved “hotspot” regions of FKS1 and FKS2, which decrease drug binding and have been observed more frequently in clinical *C. glabrata* isolates, particularly following previous echinocandin exposure (Garcia-Effron et al., 2009; Katiyar et al., 2012; Zajac et al., 2025).

#### 2.2.4 Biofilm-mediated tolerance

On catheters, prostheses, and mucosa, *C. glabrata* biofilms form dense communities embedded in an extracellular matrix (ECM) rich in extracellular DNA, proteins, and β-glucans. The matrix is responsible for creating a highly tolerant phenotype, which primarily occurs through the confinement of drugs within the matrix, thereby preventing the diffusion of drugs into the surrounding medium. Other factors contributing to high tolerance include differences in the expression of efflux pumps and stress response genes, as well as the presence of dormant (persister) cells within the matrix (Rodrigues et al., 2017; Gonçalves et al., 2021). Most importantly, fluconazole can be sequestered by the β-1,3-glucans of the matrix, which not only confers resistance to fluconazole but also provides matrix support for biofilm formation (Rodrigues et al., 2017).

Taken together, the resistance mechanisms of *C. glabrata* are not isolated adaptations but interconnected survival strategies that collectively promote multidrug resistance. These mechanisms increase fungal reliance on membrane integrity, stress-response pathways, metabolic flexibility, and biofilm architecture, thereby creating compensatory vulnerabilities. Importantly, many of these adaptations impose physiological costs that can be exploited therapeutically, particularly by agents capable of acting on multiple cellular targets simultaneously rather than a single molecular site.

Phytochemicals offer a mechanistically complementary approach to conventional antifungals. As detailed in the following sections, plant-derived compounds interfere with key resistance pathways by inhibiting efflux pumps, destabilizing membrane sterol composition, amplifying oxidative and cell wall stress, and dismantling biofilm structure (**Table 3**). Through these multitarget actions, phytochemicals can restore antifungal susceptibility, enhance drug penetration, and potentiate the activity of azoles and echinocandins against resistant *C. glabrata*. This integrated understanding of resistance biology provides the conceptual framework for evaluating phytochemical-based strategies as effective adjuncts or alternatives in the management of multidrug-resistant *C. glabrata* infections.

**Table 3:** Plant-derived compounds active against *Candida glabrata*.

Compound	Phytochemical class	Targeted resistance/Cellular mechanism	Key references
Berberine	Alkaloid (isoquinoline)	Efflux pump inhibition (Cdr1/Pdr1), mitochondrial dysfunction, azole resistance reversal	Dhamgaye et al., 2014; Gupta et al., 2023
Piperine	Alkaloid (piperidine)	Membrane modulation, metabolic interference, efflux modulation	Gupta et al., 2023
Quercetin	Flavonoid (flavonol)	Membrane disruption, ergosterol depletion, antibiofilm	Salazar-Aranda et al., 2015
EGCG	Flavonoid (catechin)	ROS induction, membrane damage, mitochondrial stress	Park et al., 2006; Chen et al., 2015
Hesperetin	Flavonoid (flavanone)	Biofilm eradication, membrane stress	Sureendar et al., 2025
Carvacrol	Terpenoid (phenolic monoterpene)	Membrane permeabilization, ergosterol disruption, antibiofilm	Ahmad et al., 2011; Soulaimani et al., 2021
Thymol	Terpenoid (phenolic monoterpene)	Membrane damage, ergosterol depletion	Ahmad et al., 2011; Balef et al., 2024
Geraniol	Terpenoid (acyclic monoterpene)	Efflux suppression (CDR1), apoptosis, ECM reduction	Gupta and Poluri, 2021
Gallic acid	Phenolic acid	Protein denaturation, ROS induction, biofilm inhibition	Teodoro et al., 2015; Alves et al., 2014
Ellagic acid	Polyphenol	Ergosterol inhibition, efflux pump suppression, redox imbalance	Sampaio et al., 2021
Oregano EO	Essential oil (carvacrol/thymol-rich)	Membrane disruption driven by carvacrol/thymol	Szweda et al., 2015; Fernandes et al., 2023
Cinnamon EO	Essential oil (cinnamaldehyde-rich)	Ergosterol inhibition, efflux suppression	Shreaz et al., 2011; Saracino et al., 2022

## 3 Essential oil and phytochemicals with antifungal activity against *C. glabrata*

### 3.1 Essential oils

Essential oils (EOs) are volatile, aromatic secondary metabolites derived from various plant parts and are composed primarily of terpenoids, phenylpropanoids, and other low-molecular-weight compounds (Butnariu, 2021). Although EOs are frequently discussed as unified antifungal agents, their antifungal activity against *C. glabrata* is largely driven by a limited number of dominant constituents rather than the oil as a whole. The relative abundance and chemical nature of these major components, such as monoterpenes and aldehydes, largely determine antifungal potency, spectrum of activity, and toxicity (Bakkali et al., 2008).

The antifungal effects of EOs against *C. glabrata* primarily arise from membrane-active constituents, particularly carvacrol, thymol, and cinnamaldehyde, which readily partition into fungal lipid bilayers. These compounds increase membrane fluidity, disrupt ergosterol-dependent integrity, and cause leakage of intracellular ions, proteins, and nucleotides, leading to rapid fungistatic or fungicidal effects (Nazzaro et al., 2013; Tyagi and Malik, 2010). In addition, dominant EO constituents can induce intracellular reactive oxygen species (ROS) accumulation or impair fungal antioxidant defense systems, resulting in mitochondrial dysfunction and apoptosis-like cell death (Boukhatem et al., 2014).

Beyond membrane disruption, specific EO constituents interfere with biofilm-associated signaling pathways, including adhesion molecules and quorum-sensing systems, thereby inhibiting biofilm formation and destabilizing established biofilms. This effect is particularly relevant for *C. glabrata*, whose biofilm-associated drug tolerance contributes significantly to clinical treatment failure (Fernández-Calderón et al., 2021; Karpiński et al., 2023). Importantly, these anti-biofilm effects are not universal across all EOs but correlate strongly with the presence and concentration of key active molecules.

Among the most studied examples, oregano oil owes its antifungal efficacy primarily to carvacrol and thymol, which exhibit strong membrane-disruptive activity and significant synergy with azoles. Studies have shown that these constituents reduce fluconazole and itraconazole MICs and enhance antifungal susceptibility in azole-resistant *C. glabrata* isolates (Szweda et al., 2015; Fernandes et al., 2023; Váczi et al., 2024; Massa et al., 2018). Similarly, the antifungal activity of cinnamon oil is largely attributable to cinnamaldehyde, which inhibits ergosterol biosynthesis, suppresses efflux pump activity, and downregulates virulence-associated pathways, thereby restoring azole sensitivity in resistant strains (Shreaz et al., 2011; Saracino et al., 2022).

Despite promising *in vitro* and preclinical findings, the antifungal efficacy of EOs should not be generalized, as activity varies widely depending on chemical composition, extraction method, and constituent ratios. Moreover, the same membrane-active properties that damage fungal cells raise concerns regarding host cytotoxicity, including skin irritation, mucosal toxicity, and potential systemic effects at higher concentrations. Additional challenges, such as volatility, poor aqueous solubility, chemical instability, and batch-to-batch variability, complicate standardization, dosing, and clinical translation (Bakkali et al., 2008; Di Vito et al., 2023; Tyagi and Malik, 2010; Weisany et al., 2022; Wicaksono et al., 2020).

### 3.2 Flavonoids

Flavonoids are a broad class of polyphenolic phytochemicals widely distributed in vegetables, fruits, and medicinal plants. Although frequently discussed as a single group, antifungal activity among flavonoids is highly compound-dependent and should not be generalized across the class (Al Aboody and Mickymaray, 2020). At the cellular level, several flavonoids exert antifungal effects through membrane-targeting mechanisms, including increased membrane permeability, leakage of cytoplasmic contents, and destabilization of lipid organization. Flavonols such as quercetin and luteolin have been shown to disrupt fungal membrane integrity and permeability, resulting in loss of cell viability (Salazar-Aranda et al., 2015; Al Aboody and Mickymaray, 2020). Some flavonoids also inhibit ergosterol biosynthesis, leading to altered membrane composition and increased susceptibility to environmental and drug-induced stress (Dantas et al., 2025). In parallel, compounds such as quercetin and catechins can induce oxidative stress, overwhelming fungal antioxidant defenses and triggering apoptosis-like or necrotic cell death pathways (Stachelska et al., 2025; Tóth Hervay et al., 2024).

Quercetin is among the most consistently studied flavonoids against *Candida* spp. While much of the mechanistic work has been conducted in *C. albicans*, quercetin has demonstrated inhibitory activity against *C. glabrata* planktonic cells, with MICs comparable to fluconazole in some studies. Importantly, quercetin suppresses biofilm formation, downregulates virulence-associated genes, and enhances azole susceptibility, suggesting translational relevance for *C. glabrata*, which shares conserved efflux- and biofilm-associated resistance mechanisms (Salazar-Aranda et al., 2015; Schwarz et al., 2022).

Among catechins, epigallocatechin-3-gallate (EGCG) from green tea exhibits particularly strong intrinsic antifungal activity against *C. glabrata*. Under standardized EUCAST conditions, EGCG displayed a markedly lower MIC (0.3125 µg/mL) compared with fluconazole (4.0 µg/mL), and showed fungicidal effects at higher concentrations (Chen et al., 2015). These findings position EGCG as one of the most potent naturally occurring flavonoids against *C. glabrata in vitro*, although its clinical applicability is constrained by pharmacokinetic limitations.

Hesperetin has emerged as a notable antibiofilm agent. In clinical *C. glabrata* isolates, hesperetin eradicated mature biofilms at 2× MIC, achieving near-complete loss of viability within 24 hours, highlighting its potential relevance for biofilm-associated infections that are refractory to conventional antifungals (Sureendar et al., 2025).

Other flavonoids, including kaempferol, myricetin, luteolin, fisetin, and baicalein, show variable but measurable antifungal activity against *C. glabrata*, with MICs ranging from low single-digit µg/mL to values comparable with fluconazole (Salazar-Aranda et al., 2015). This variability reinforces the necessity of compound-level evaluation rather than class-wide extrapolation.

Beyond natural flavonoids, chalcone derivatives and semi-synthetic flavonoids demonstrate enhanced antifungal potency. Isoquercitrin exhibited consistent synergy with isavuconazole against resistant *C. glabrata* isolates (FICI 0.125–0.5) (Schwarz et al., 2022). Synthetic chalcone–triazole hybrids and benzofuran–indole chalcones inhibited *C. glabrata* growth at concentrations ≤50 µg/mL, while a brominated flavonoid derivative (BrCl-flav) outperformed fluconazole against resistant strains (Babii et al., 2021; Singampalli et al., 2025).

Despite strong *in vitro* efficacy, the clinical translation of flavonoids remains limited by poor aqueous solubility, low oral bioavailability, rapid metabolism, and restricted systemic exposure. Consequently, future work should prioritize drug delivery strategies, structural optimization, and *in vivo* pharmacokinetic studies to fully realize the therapeutic potential of compound-specific flavonoids against *C. glabrata* infections.

### 3.3 Terpenoids

Of all secondary metabolites produced by plants, terpenoids, including monoterpenes, sesquiterpenes, diterpenes, and triterpenoids, exhibit the greatest structural diversity (Ashour et al., 2010). Their pronounced hydrophobicity enables strong interactions with fungal cell membranes and intracellular targets, contributing to their antimicrobial potential (Huang et al., 2022). There is growing interest in terpenoids as antifungal or antifungal-adjuvant agents against *C. glabrata*, particularly due to their capacity to penetrate biofilms and circumvent resistance mechanisms such as efflux pump overexpression and alterations in ergosterol biosynthesis (Haque et al., 2016; Ivanov et al., 2022). Mechanistically, terpenoids exert antifungal effects through membrane disruption, interference with cell wall assembly, mitochondrial dysfunction, and modulation of oxidative stress pathways (Haque et al., 2016; Ivanov et al., 2022).

#### 3.3.1 Highly active monoterpenes

Among terpenoids, phenolic monoterpenes exhibit the most potent and consistent antifungal activity against *C. glabrata*. Carvacrol and thymol are the best-characterized examples, demonstrating strong activity against both planktonic cells and biofilms. These compounds insert into the lipid bilayer, reducing membrane fluidity, increasing permeability, and causing leakage of ions and intracellular contents (Ahmad et al., 2011; Rao et al., 2010). This nonspecific membrane-disruptive mechanism is particularly advantageous against *C. glabrata*, whose resistance is often mediated by efflux pumps and ergosterol pathway alterations. Thymol additionally reduces ergosterol levels, while carvacrol inhibits fungal adhesion and disrupts established biofilms, frequently showing synergy with fluconazole (Ahmad et al., 2011; Kauser et al., 2024; Soulaimani et al., 2021; Balef et al., 2024).

Geraniol is another highly active monoterpene with robust antibiofilm efficacy against *C. glabrata*. Geraniol inhibits planktonic growth and eradicates mature biofilms by reducing extracellular matrix carbohydrates and eDNA, downregulating efflux pump (*CDR1*) and ergosterol biosynthesis genes, and inducing apoptosis-like cell death (Gupta and Poluri, 2021). Perillyl alcohol similarly disrupts biofilm biomass, perturbs ergosterol content, damages cell wall and membrane integrity, and enhances azole efficacy, allowing reduced antifungal dosing (Gupta and Poluri, 2021; Ansari et al., 2016).

Other active monoterpenes include eugenol and linalool, both of which exhibit measurable MIC and MFC values against clinical *C. glabrata* isolates. Eugenol and its synthetic glucoside derivatives display antifungal activity in the micromolar range while maintaining relative selectivity toward fungal cells over mammalian cells (De Souza et al., 2016; Didehdar et al., 2022). Linalool also shows synergistic effects with antifungal and antiseptic agents in checkerboard assays (Biernasiuk and Malm, 2023).

#### 3.3.2 Weakly active terpenoids

In contrast, not all monoterpenes exhibit strong antifungal activity. Limonene, a non-phenolic monoterpene abundant in citrus essential oils, shows relatively weak and primarily fungistatic effects against *Candida* spp. While limonene can inhibit fungal growth at higher concentrations, it lacks potent fungicidal or antibiofilm activity compared with phenolic monoterpenes such as carvacrol, thymol, and geraniol (Ahmedi et al., 2024). These findings underscore that antifungal efficacy among terpenoids is structure-dependent and cannot be generalized across the class.

#### 3.3.3 Triterpenoids

Beyond monoterpenes, triterpenoids such as ursolic acid and oleanolic acid represent a mechanistically distinct group with antifungal potential. Ursolic acid disrupts membrane integrity, inhibits efflux pumps, and induces oxidative stress in *Candida* species, including *C. glabrata* (Marena et al., 2021). Oleanolic acid, a structural analog, may inhibit ergosterol biosynthesis and reduce biofilm biomass (Tan et al., 2024). Although these compounds typically display higher MIC values than phenolic monoterpenes, their multitarget activity highlights their potential as lead scaffolds for antifungal development.

Essential oils rich in carvacrol or thymol, such as those from *Origanum vulgare* and *Thymus vulgaris*, demonstrate fungicidal activity against multiple *Candida* species, including *C. glabrata*. However, antifungal efficacy varies with chemical composition and strain origin, emphasizing the importance of attributing activity to specific constituents rather than essential oils as homogeneous mixtures (Cleff et al., 2010; Vahedi et al., 2016).

### 3.4 Phenolic compounds

Phenolic compounds represent a large and structurally diverse group of plant secondary metabolites, ranging from simple phenols and phenolic acids to complex polyphenols such as tannins (Crozier et al., 2006). Their chemical diversity enables interaction with multiple fungal targets, making phenolics attractive candidates for combating drug-resistant fungal pathogens, including *C. glabrata*. However, antifungal efficacy among phenolic compounds is highly compound-dependent and cannot be generalized across the class. Instead, differences in molecular complexity strongly influence potency, mechanism of action, and relevance for therapeutic development.

#### 3.4.1 Simple phenols and phenolic acids

Simple phenolic compounds, including caffeic acid and gallic acid, exert antifungal activity primarily through protein denaturation and enzymatic inhibition, leading to disruption of essential metabolic pathways (Teodoro et al., 2015). These compounds interact directly with fungal proteins, impairing enzymatic catalysis and reducing cellular viability. In addition, simple phenolics disrupt mitochondrial function by interfering with the electron transport chain, resulting in ATP depletion, an effect that is particularly detrimental to *C. glabrata*, which relies heavily on metabolic plasticity for survival (Hassan et al., 2021; Salazar-Aranda et al., 2015; Alves et al., 2014).

Although phenolic acids are widely recognized as antioxidants in mammalian systems, their antifungal activity is predominantly associated with pro-oxidant effects within fungal cells. Gallic acid, in particular, induces intracellular ROS accumulation, disrupts redox homeostasis, and compromises mitochondrial integrity, ultimately leading to fungal cell death (Teodoro et al., 2015; Alves et al., 2014). Gallic acid has also been shown to increase membrane permeability, promote leakage of intracellular contents, reduce biofilm biomass, and enhance susceptibility to azoles, although its intrinsic antifungal potency is generally moderate and concentration-dependent.

#### 3.4.2 Complex polyphenols

In contrast to simple phenolic acids, ellagic acid, a complex polyphenol abundant in pomegranates, berries, and nuts, exhibits broader and more targeted antifungal activity against *C. glabrata*. Ellagic acid acts through multiple resistance-relevant mechanisms, including inhibition of ergosterol biosynthesis and suppression of efflux pump activity, both of which are central to azole resistance in *C. glabrata* (Sampaio et al., 2021; Wicaksono et al., 2020). Importantly, ellagic acid has been shown to restore azole susceptibility in resistant *C. glabrata* isolates, positioning it as a promising antifungal adjuvant rather than a standalone agent.

Similar to gallic acid, the antifungal efficacy of ellagic acid is not attributable solely to antioxidant activity. Instead, its ability to induce redox imbalance, disrupt membrane integrity, and interfere with resistance-associated pathways underlies its antifungal action. The simultaneous targeting of membrane composition, efflux systems, and oxidative stress responses confers ellagic acid a higher functional relevance compared with simpler phenols.

#### 3.4.3 Virulence and biofilm modulation

Beyond direct fungistatic or fungicidal effects, phenolic compounds can attenuate *C. glabrata* virulence by inhibiting adhesion, suppressing hydrolytic enzyme activity, and disrupting biofilm formation. Several phenolics downregulate genes involved in adhesion and extracellular matrix production, resulting in reduced biofilm biomass and increased susceptibility to antifungal therapy (Hassan et al., 2021; Rodrigues et al., 2017; Kumar and Kumar, 2023; Gupta et al., 2018; Duggan and Usher, 2023). These antivirulence effects are particularly relevant for *C. glabrata*, where biofilm-associated resistance contributes substantially to treatment failure.

### 3.5 Alkaloids

Alkaloids are chemically diverse nitrogen-containing secondary metabolites produced by numerous plant species, many of which have a long history of use in traditional medicine and documented antimicrobial activity (Thawabteh et al., 2024; Gupta et al., 2023). Although alkaloids are often discussed as a single antifungal class, their activity against *C. glabrata* is highly compound-specific and cannot be generalized. Only a limited number of alkaloids have been experimentally validated against *C. glabrata*, while many others lack sufficient activity or selectivity.

Among alkaloids, berberine is the most extensively characterized compound with confirmed antifungal relevance to *C. glabrata*. Berberine, isolated from *Berberis*, *Coptis*, and *Hydrastis* species, exhibits moderate intrinsic antifungal activity (reported MICs typically in the range of 64-256 µg/mL) but demonstrates pronounced synergistic effects with azole antifungals. Mechanistic studies have shown that berberine inhibits ABC efflux pumps, particularly Cdr1 through Pdr1-regulated pathways, disrupts mitochondrial function, and increases intracellular accumulation of fluconazole, leading to reversal of azole resistance in *C. glabrata*. Synergy with fluconazole has been confirmed in checkerboard assays, with reported fractional inhibitory concentration index (FICI) values ≤ 0.5, indicating true pharmacological synergy rather than additive effects (Dhamgaye et al., 2014; Gupta et al., 2023; Yong et al., 2020; Tong et al., 2021; Zheng et al., 2023).

Piperine, the principal alkaloid of *Piper nigrum*, exhibits limited standalone antifungal activity and should not be considered a primary antifungal agent. Instead, piperine functions predominantly as an adjuvant compound, enhancing antifungal efficacy through modulation of membrane dynamics, interference with drug efflux, and perturbation of fungal metabolism. While MIC/MFC values against *C. glabrata* are not consistently reported, available data suggest that piperine improves susceptibility to conventional antifungals rather than exerting strong fungicidal activity on its own.

Importantly, many plant-derived alkaloids frequently cited for antifungal properties, particularly those studied in *C. albicans* or other fungal species, have not been validated against *C. glabrata*. Current evidence supports antifungal relevance only for a small subset of alkaloids, with berberine representing the most robust example.

In addition to efficacy considerations, alkaloids warrant cautious evaluation due to host toxicity and drug–drug interaction risks. Several alkaloids induce dose-dependent adverse effects, including hepatotoxicity, neurotoxicity, cardiotoxicity, and cytotoxicity, largely through interactions with nucleic acids, mitochondrial function, and ion channels. Moreover, alkaloids can modulate drug-metabolizing enzymes and transporters, including cytochrome P450 isoenzymes (e.g., CYP3A4 and CYP2D6), UDP-glucuronosyltransferases, and P-glycoprotein, thereby altering the pharmacokinetics of co-administered drugs (Zhou, 2008). Berberine has been specifically reported to inhibit CYP3A4 and CYP2D6, raising the possibility of clinically relevant interactions when used alongside antifungals or other therapeutics (Gupta et al., 2023).

## 4 Synergistic interactions with conventional antifungals

A synergistic interaction of plant-based phytochemicals alongside traditional antifungal agents is becoming more prevalent in the treatment of MDR *C. glabrata*. This species is capable of overexpressing efflux pumps, forming biofilms, and developing resistance quickly. Therefore, many of the medications currently available, especially the azoles, are less effective. However, phytochemicals enhance susceptibility to antifungal agents via targeting different pathways, thus weakening fungal defenses and increasing the amount of drug delivered into the cell (De Andrade Monteiro and Ribeiro Alves Dos Santos, 2020). One of the most well-recognized mechanisms is efflux pump inhibition, as compounds such as quercetin and berberine have been shown to reduce the activity of ABC transporters like Cdr1 and Cdr2, restoring intracellular concentrations of fluconazole in resistant *C. glabrata* strains (Gupta et al., 2018; Ding et al., 2025). Membrane-active agents such as thymol, carvacrol, and several flavonoids disrupt the fungal plasma membrane, resulting in increased permeability to both azoles and polyenes (Kumar et al., 2019; Jadimurthy et al., 2023; Al Aboody and Mickymaray, 2020).

Another significant aspect of how synergism occurs includes modulation of the ergosterol pathway. Compounds that inhibit or decrease levels of ergosterol can improve binding of azoles to lanosterol 14α-demethylase (an enzyme targeted by azoles) and thus enhance the antifungal activity of azoles (Siswina et al., 2023). Additionally, many phytochemicals have been demonstrated to exhibit strong anti-biofilm effects. Cinnamaldehyde, EGCG, and apigenin can increase the susceptibility of *C. glabrata* biofilms to treatment with fluconazole and echinocandins due to their ability to disrupt components of the biofilm, such as the ECM and the adhesion of the fungal cells (Fydrych et al., 2025; Shariati et al., 2022; Bonincontro et al., 2023). These studies are important because biofilm-mediated antifungal tolerance is a significant obstacle to overcoming resistance to antifungal therapy in the clinical setting.

Despite these promising findings, a key limitation across the current literature is the absence of a standardized framework for assessing antifungal synergy. While checkerboard assays and time-kill studies are frequently employed, FICI values are not consistently reported, or different interpretive cut-offs are used, which hampers direct comparison between studies and limits translational relevance (De Andrade Monteiro and Ribeiro Alves Dos Santos, 2020). Standardized reporting of synergy metrics, including clearly defined FICI thresholds, strain backgrounds, and experimental conditions, is therefore essential to strengthen the rigor and reproducibility of phytochemical-antifungal combination studies.

Hopefully, the synergy created by combining phytochemicals with traditional antifungal drugs will help address *C. glabrata*’s MDR phenotype. The use of multiple physiologically active agents simultaneously, targeting various issues such as efflux, cell membrane integrity, stress response, and biofilm structure, should allow for restoration of the susceptibility of *C. glabrata* to antifungal drugs, enabling a decrease in the amount needed to treat, as well as potentially decreasing the risk of toxicity due to enhanced dosages of antifungal drugs. Continued studies *in vitro*, *in vivo*, and in the clinic are warranted, as the prevalence of drug resistance continues to rise worldwide, and the synergy achieved through these combinations of phytochemicals and antifungal drugs represents an exciting possibility for future therapeutic development.

## 5 Challenges and future perspectives

There have been numerous studies and advancements in identifying and researching the potential of phytochemicals as antifungal agents for *C. glabrata*; however, several barriers still prevent the use of these compounds in clinical settings. The primary barrier to clinical use is the variability in the composition of phytochemicals due to differences between plants, including but not limited to; species type; geographic location; the conditions under which the plant was grown; the extraction methods utilized; and the time at which it was harvested; all of these factors affect the presence/amount of, and the uniformity of, the bioactive compounds contained in the plant (Cowan, 1999; Scalbert et al., 2005). The inherent variability of phytochemical composition makes it increasingly challenging to achieve standardized products, batch-to-batch comparability, and regulatory approval for plant-derived antifungal therapies. To meet regulatory expectations for antifungal drug development, phytochemicals must undergo rigorous chemical characterization, including validated fingerprinting, identification of active constituents, and compliance with Good Manufacturing Practice standards. Without defined composition and reproducible bioactivity, advancement into preclinical and clinical development pipelines remains limited.

Even though many phytochemicals, such as phenolics, terpenoids, and alkaloids, exhibit strong *in vitro* activity, the overall lack of solid *in vivo* pharmacokinetic, pharmacodynamic, and toxicological data creates a significant obstacle to their advancement into the clinical development pipeline (Guevara-Lora et al., 2020; Didehdar et al., 2022). Additionally, there are currently very few clinical trials conducted for plant-derived antifungal agents, and no standardized methodologies exist to evaluate synergism, biofilm formation, or development of drug resistance, which has resulted in a lack of agreement between studies (Gupta and Poluri, 2021; Esmaeili et al., 2025). From a clinical development perspective, phytochemical-based antifungal strategies require a structured translational framework beginning with validated *in vivo* efficacy and toxicity studies, followed by phase I clinical trials assessing safety, tolerability, and pharmacokinetics. Subsequent phase II studies should focus on efficacy, particularly in combination with existing azoles or echinocandins, given the proposed role of phytochemicals as resistance-modifying or adjunctive agents in MDR *C. glabrata* infections. Interactions between antifungal medications and phytochemicals require thorough examination for potential synergism or antagonism with currently available antifungals, particularly with azole antifungals, and their subsequent impact on therapeutic outcomes (Soulaimani et al., 2021; Katragkou et al., 2015). Standardized methodologies for evaluating synergism, biofilm disruption, and resistance suppression should be prioritized to ensure cross-study comparability and regulatory acceptance of combination therapies.

Emerging research avenues will be necessary to address the challenges faced when developing phytochemicals against MDR *C. glabrata*. Advanced omics technologies, including transcriptomic, metabolomic, proteomic, and lipidomic approaches, can enhance understanding of the underlying mechanisms through which phytochemicals impact the action of efflux pumps, stress response pathways, virulence factors, and biofilm structure of *C. glabrata* (Silva et al., 2017; Rodrigues, 2018; Tits et al., 2020). Developing reliable chemical fingerprinting and extraction-standardization protocols will be essential for generating reproducible, quantifiable, and compliant (regulatory) phytochemical profiles for research and approval. Synergistic formulations combining phytochemicals with conventional antifungals represent a particularly promising strategy, as they may restore azole or echinocandin susceptibility, reduce required drug dosages, and limit the emergence of further resistance (Tits et al., 2020; Ansari et al., 2016; Nikoomanesh et al., 2023). Biological delivery systems made using nanotechnology, such as liposomes, nanoparticles, and nanoemulsions, offer exceptional solutions for enhancing: The effectiveness and stability of phytochemicals, along with the ability to improve targeted delivery of phytochemicals to deep tissue and the biofilm structure (Khoee and Madadi, 2023; De Almeida Campos et al., 2023). The incorporation of standardized *in vivo* models of mucosal, systemic, and biofilm-associated *C. glabrata* infections will be essential for bridging the gap between *in vitro* findings and clinical relevance, and for generating data suitable for regulatory submission (Hassan et al., 2021). Interdisciplinary collaboration amongst microbiologists, pharmacognosists, nanoscientists, chemists, and clinical researchers will be necessary to develop viable treatment options using phytochemicals for the treatment of MDR *C. glabrata*.

## 6 Conclusion

The increasing prevalence of MDR *C. glabrata* represents a significant challenge for current antifungal therapy and underscores the need for continued research into alternative and adjunctive treatment strategies. Phytochemicals encompass a diverse range of structural classes and exhibit multiple antifungal mechanisms, including disruption of fungal membranes, interference with ergosterol biosynthesis, modulation of oxidative stress, inhibition of drug efflux pumps, reduction of adhesion, and inhibition of biofilm formation. Among the most compelling strategies to overcome these barriers is the exploration of synergistic mechanisms between phytocompounds and conventional antifungals. Evidence suggests that phytochemicals such as phenolics, terpenoids, and alkaloids can potentiate the activity of azoles and echinocandins by modulating efflux pump activity, disrupting biofilm architecture, and attenuating virulence pathways. Such synergy not only enhances therapeutic efficacy but also reduces required drug dosages, thereby minimizing toxicity and delaying the emergence of resistance. Moreover, these interactions are variable and largely supported by *in vitro* studies, with effects broadly comparable to those reported for other *Candida* species. Significant challenges remain, including variability in phytochemical composition, limited pharmacokinetic and toxicological data, and the scarcity of well-designed *in vivo* and clinical studies. Future progress will depend on standardized extraction methods, mechanistic studies using systems biology approaches, and rigorous preclinical and clinical evaluation to determine whether phytochemical-based strategies can be reliably integrated into antifungal therapy for MDR *C. glabrata*.
